# Adapting a pregnancy app (HealthyMoms) to support healthy habits in migrant women—a qualitative study on women's preferences and perceived needs to support health behaviors during pregnancy

**DOI:** 10.1177/20552076241304045

**Published:** 2024-12-12

**Authors:** Emmie Söderström, Christina Alexandrou, Sara Bressanutti, Johanna Sandborg, Anna-Karin Lindqvist, Marie Löf

**Affiliations:** 1 Department of Health, Medicine and Caring Sciences, 4566 Linköping University, Linköping, Sweden; 2 Department of Medicine, Karolinska Institutet, Huddinge, Stockholm, Sweden; 3 Department of Psychobiology, 16781 University of Valencia, Valencia, Spain; 4 Division of Health, Medicine and Rehabilitation, Department of Health, Education and Technology, 5185 Luleå University of Technology, Luleå, Sweden

**Keywords:** mHealth, telemedicine, migrant, healthcare, maternity care, pregnancy, qualitative research methods, healthy lifestyle behaviors

## Abstract

**Background:**

Healthy lifestyle behaviors in pregnancy are important for maternal and offspring health. Mobile health (mHealth) tools have potential to provide support for lifestyle behaviors at scale but such tools are commonly developed only in native languages, limiting reach in migrant populations.

**Objectives:**

This qualitative study, in two of the largest migrant populations in Sweden (Arabic- and Somali-speaking women), aimed to explore (a) perceptions and needs of knowledge and support related to pregnancy and lifestyle behaviors and (b) needs of and attitudes towards a pregnancy app and how features and content in the app can be adapted to support healthier lifestyle behaviors in migrant women.

**Method:**

Individual semi-structured interviews were conducted with Arabic (*n* = 10) and Somali-speaking women (*n* = 9). Data was analyzed using content analysis (inductive latent approach).

**Results:**

Arabic- and Somali-speaking women described an increased need of knowledge regarding pregnancy and lifestyle behaviors. The social environment could both motivate behavior change and be a source of misinformation. Women expressed great trust in maternity healthcare but requested more information related to lifestyle behaviors. A pregnancy app was perceived as a helpful tool to support healthy lifestyle behaviors. Mere translations were suggested to be valuable, however, audio- and video-format to deliver content and inclusion of women's partners in the app were described as desirable adaptations.

**Conclusion:**

Our findings can guide maternity healthcare on what support migrant women need and inform future development of mHealth tools for pregnant migrant populations. Future research should disentangle the degree of cultural adaptations required for mHealth lifestyle interventions provided by healthcare.

## Introduction

Optimal nutrition and physical activity are essential during pregnancy and should consequently be a key element of maternity routine care. Topics to address in maternity healthcare include the increased physiological requirements of energy and nutrients^1–4^ but also support to avoid excessive gestational weight gain (GWG), which increases risks for pregnancy complications such as gestational diabetes mellitus, large-for-gestational-age infants, and postpartum weight retention.^5,6^ Providing support for a healthy lifestyle in pregnancy has become even more important considering that the global prevalence of obesity has significantly increased in the past decades,^7^ which also applies to pregnant women.^8,9^ For instance, pre-pregnancy obesity has been reported to continue to increase in the US, reaching levels as high as 20–36%.^10^ In Sweden, the prevalence of obesity in pregnant women at admission to maternity healthcare has tripled between 1992 and 2022, now concerning almost 1 in 5 women.^11^ Furthermore, health inequalities are observed in high-income countries, where increased levels of excessive GWG, obesity and obesity-associated pregnancy complications (e.g., gestational diabetes mellitus) are reported to be more common among socially disadvantaged and migrant populations.^10,12,13^ Consequently, evidence-based, low-cost and scalable tools, that can assist maternity healthcare in their work to support healthy eating and physical activity during pregnancy, are urgently required. It is essential that such tools are inclusive and accessible *for all,* irrespectively of language or health literacy level, however, such tools are lacking but are being called for.^14^

Advancements in digital technologies enable new opportunities for health promotion in maternity healthcare. One such technology, mobile health (mHealth), refers to the use of mobile and wireless devices (mobile phones, tablets, wearable sensors etc.) with advantages such as real-time monitoring and feedback, less burden on healthcare as well as cost-effectiveness.^15^ mHealth has for instance been successfully integrated into interventions to promote healthy diets, physical activity, and weight loss.^16,17^ We have previously showed that a comprehensive lifestyle and pregnancy intervention grounded in social cognitive theory and including key behavior change techniques delivered through a smartphone app (*HealthyMoms*) successfully improved dietary habits and lowered GWG in healthy Swedish-speaking women during pregnancy^18^ and the app was further appreciated by the end-users.^19^ Given the positive effect of the app and its feasibility, it has potential to be implemented at scale in Swedish maternity healthcare. However, given the fact that 20% of the population in Sweden is foreign-born,^20^ the app needs to be adapted to other languages to ascertain its inclusiveness and accessibility *for all*. In relation to this, it has previously been emphasized that more data is needed on migrant populations needs and access to healthcare, and that research is lacking regarding migrant populations’ own views of this topic.^21^ In addition, there is a need of exploring migrant populations’ perceived needs of mHealth solutions as well as what cultural adaptation in such tools are required, which indeed is an emerging research area.^22,23^ We have previously conducted pioneering research in this area as part of the MoBILE research program.^24^ For instance, we translated and adapted an app (MINISTOP) for obesity prevention in families with young children, into Somali and Arabic.^25^ This work was based on qualitative research in close collaboration with families, making sure that the content was culturally adapted to food traditions and other relevant aspects in addition to translation. As a result, we managed to reach children with foreign-born parents at similar rates as the general population is Sweden.^26^ The app was in addition highly appreciated by Arabic and Somali-speaking families and supported behavior change.^25^ Thus, we have previously shown that it is possible to increase reach with translations and adaptations of content in digital prevention interventions. This potential has yet to be explored for promotion of healthier eating, physical activity and GWG among pregnant women.

Thus, we sought out to explore views among two of the largest migrant groups in Sweden, i.e., women from Arabic- and Somali-speaking countries,^27^ more specifically we aimed to explore (a) perceptions and needs of knowledge and support related to pregnancy and lifestyle behaviors and (b) needs of, and attitudes towards, a pregnancy app and how features and content in the app can be adapted to support healthier lifestyle behaviors in migrant women.

## Material and methods

### Participants

This study utilized a qualitative study design with individual interviews with foreign-born women (from Arabic- and Somali-speaking countries) residing in Sweden. Participants were purposively recruited through maternity healthcare in the county of Östergötland in Sweden (October 2020 to August 2021). Recruitment took place during group meetings with a midwife and interpreter present, where also one member of the research team (ES) attended to inform about the study. In addition, midwives informed potential participants about the study during routine individual visits. Pregnant women aged 18 years or older, born in an Arabic- or Somali-speaking country were eligible for participation. Women that expressed interest in participating received full study information (in their preferred language) and had the possibility to ask questions during a phone call with an Arabic- or Somali-speaking person that collaborated with the research team. The women provided their written informed consent prior to the interview. Sampling continued until a rich material was obtained with perspectives from both Arabic- and Somali- speaking women. A total of 35 women expressed interest in participating in the study, 16 women later declined or did not reply when being contacted to receive complete study information. Thus, a total of 19 women completed the interviews (10 Arabic-speaking [9 with an interpreter] and 9 Somali-speaking women [6 with an interpreter]). The Consolidated criteria for REporting Qualitative Research (COREQ) checklist^28^ was applied and all study procedures were approved by the Swedish Ethical Review Authority (Reference number: 2020-01447).

### Data collection

All interviews were conducted over telephone and a three-way-call was used for interviews with an interpreter (either in Somali or Arabic). The interpreters were hired by the research team specifically for this study. Interviews were conducted using a semi-structured interview guide (Supplementary file 1) that had been developed by the research team with training and knowledge in nutrition, qualitative- and mHealth research, migrant health and with knowledge and insights into maternity healthcare. The guide was compiled of core questions regarding women's experiences of pregnancy and maternity healthcare, lifestyle behaviors and thoughts about a pregnancy app, more specifically the previously evaluated *HealthyMoms* app for Swedish-speaking women^18^ and how it potentially could be adapted to non-Swedish speaking women (e.g., in terms of food culture and other relevant aspects). The questions in the interview guide were based on previous research from the research group that been conducted in pregnant and migrant populations.^19,29^ In order for participants to get an understanding of the features of the HealthyMoms app, a clear description of the content was provided during the interviews (including information on overall content of themes delivered every second week,physical activity including exercise videos, recipe section, pregnancy calendar and registration functions for diet, physical activity and GWG, and feedback functions based on registered data). The interviewer followed up the individual responses with probing questions when needed and wrote field notes during the interviews. The interview guide was pilot tested, and the pilot interview (*n* = 1) was also included in the analysis, and only smaller changes to the interview guide were made, e.g., how some of the questions were asked. All interviews were conducted by the first author (ES), a female PhD student and nutritionist with basic education and training in qualitative methodology and previous experience from conducting qualitative interviews (e.g.,^30,31^). There was no relationship established prior to the interviews, nor did the participants have knowledge about the interviewer. Recruitment of participants and data collection continued until data saturation was reached, i.e., when broad and rich data to answer the study aim had been obtained. Interviews were audio recorded (average duration was 69 min, range: 45–105 min), fully transcribed by a professional transcription company, and transcriptions were then checked by ES. In conjunction with the interviews, the women also answered a brief questionnaire (developed by the research team) regarding their age, country of birth, education level, parity and time living in Sweden (Supplementary file 1). The participating women were on average 29 (SD 6) years old (range 20–41 years), 63% (*n* = 12) were nulliparous and participants covered a wide range of gestational weeks (14–40 weeks), although the majority were in their third trimester. The sample included women that recently had come to Sweden as well as those who had been living in the country for a longer period (time in Sweden: 7 [SD 6] years; range 0.5–18 years). Five women had a university degree (26%), while the remaining had finished elementary school (*n* = 6, 32%) or high school (*n* = 4, 21%) or had not gone to school (*n* = 3, 16%). One participant did not report her educational background. The Arabic-speaking women were born in Syria (*n* = 3), Iraq (*n* = 5), Sudan (*n* = 1), and Eritrea (*n* = 1).

### Data analysis

We analyzed transcribed data using content analysis by means of an inductive latent approach with inspiration from Graneheim and Lundman.^32,33^ The following procedures were executed: after listening to all the interviews and reading the transcripts, ES and a PhD student and psychologist (SB), performed initial manifest coding using a data driven and iterative process with the research aims taken into consideration. ES proceeded to conduct latent coding to find underlying meaning. ES and SB then further condensed the codes into preliminary categories and together discussed these until consensus on the categories was reached. ES explored patterns in the data and organized the preliminary categories into potential themes. ES, CA, SB, JS and ML reviewed and refined the themes until consensus was reached regarding their content and labelling. AKL, researcher with extensive expertise in qualitative methodology, reviewed and provided feedback on the final themes.

## Results

**Figure 1.**
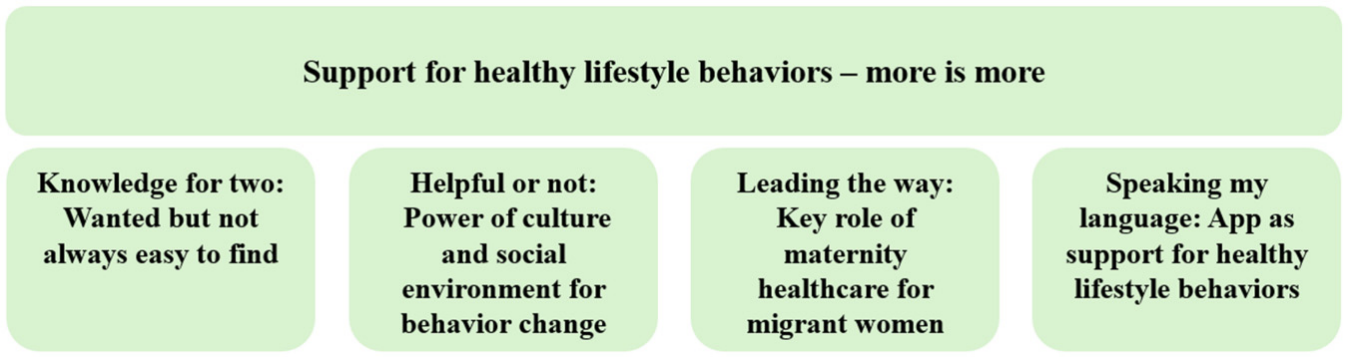
Identified themes from content analysis.

This study aimed to explore migrant women's knowledge of pregnancy and healthy lifestyle behaviors, their perceptions of the support provided by maternity healthcare as well as how a previously developed mHealth intervention (the *HealthyMoms* app) intended to support a healthy lifestyle and weight gain in pregnancy could be adapted to support them. One main theme and four sub-themes were identified in the analysis (Figure 1). To support the theme categorization, excerpts from transcribed data which are labelled with the women's origin (Somali- or Arabic-speaking), presence of an interpreter, and the women's age are included.

### Support for healthy lifestyle behaviors—more is more

Women acknowledged a need of more knowledge related to pregnancy in general and diet and physical activity in particular. Women highlighted the importance of the social environment to be supportive for behavior change during pregnancy, but the social environment could also increase worries due to misinformation regarding lifestyle behaviors. Women expressed great trust in maternity healthcare and asked for more support regarding lifestyle behaviors. An app to increase support for lifestyle behaviors in women's native language was perceived as helpful.

### Knowledge for two: wanted but not always easy to find

Women described pregnancy as a period with increased need of knowledge and information. The need was especially prominent in women expecting their first child but was also seen in women with previous experience of pregnancy and delivery. Women that recently had come to Sweden indicated a need of building a new knowledge-foundation, given that they base their knowledge about healthcare, delivery, and pregnancy from experiences in their home country. Women voiced a common need for general pregnancy information, e.g., pregnancy-related symptoms and knowledge related to delivery. Most women had knowledge of the importance of lifestyle behaviors (i.e., healthy diet and physical activity) but did not necessarily engage in them during pregnancy and they expressed a need for more specific dietary information. There was a general awareness of what causes GWG, but women lacked knowledge about why GWG is of importance in terms of pregnancy outcomes for mother and baby and some even related excessive GWG with a healthier baby.

*This pregnancy I am very relaxed and think “No, but I can gain as much weight as I want and then I will deal with it afterwards”* **Somali woman, interview without interpreter, 24 years**

Women reported trying to control and own their knowledge by asking maternity healthcare for information. Maternity healthcare was further described to have an important role in guiding women in what information is reliable and by providing in-depth information. Women further described searching for information themselves but reported struggles in identifying credible sources, especially if being pregnant for the first time. One highlighted credible source was a national website for information (provided by healthcare). Although this website often was recommended by maternity care for pregnancy related information, women reported having difficulties navigating and understanding the content when not fluent in Swedish. This was expressed as a problem in general, and not only related to this webpage. Women also reported relying on translation services to access information from digital sources.

*Sometimes we get adviced that we should go to 1177 [healthcare page], from the midwife, that you can look for information there […] sometimes it takes time to find and then to understand the Swedish*. **Somali woman, 37 years, interview with interpreter**

Further, women described turning to their social environment if they did not know where to look for specific information or if they wanted to increase their pregnancy knowledge. Those key persons in the women's social environment often included their own mothers, other close family members or friends with experience from pregnancy and delivery.

### Helpful or not: power of culture and social environment for behavior change

Women recognized that their lifestyle behaviors were shaped by their cultural heritage and social environment. Both Arabic- and Somali-speaking women voiced that their social environment could be the difference between adopting a new healthy lifestyle behavior or not. Particularly partners could motivate women to eat healthier or to be physically active during pregnancy, especially if they were aware of the benefit for the mother and child. It was also discussed that partners should be included throughout pregnancy to get more knowledge and thereby also be able to provide more support for women.

*Myself, I don't know, I don't think I would walk that much, I have my husband here, he is the one who pushes me “come on, we're going for a walk tonight” […] he motivates me, it would make it easier if you have someone who, for example, supports you with things like that*. **Somali woman, 27 years, interview without interpreter**

In contrast, women also voiced that family members and peers could influence women by giving them advice that contradict recommendations from healthcare. Such misinformation was linked to diet but mostly physical activity, where a common belief was that physical activity should be avoided to not harm the baby or mother and some women reported avoiding physical activity due to such misinformation. Women expressed that they understood that this information had been passed down for generations in their home country and most women expressed resilience against such misinformation. However, women also reported confusion when experiencing contrasting recommendations, i.e., from maternity healthcare versus advice from the social environment.

*[…] Sometimes it can be a bit much to be told “But what are you doing? Don't bend over too much, you're pregnant.” “Yes, but what am I going to…?” I also can't change my life just because I'm pregnant*. **Somali woman, 24 years, interview without interpreter**

Women also described that pregnancy could be a golden opportunity for long-lasting behavior change, especially if women could see the family benefit from it, e.g., if children increased their vegetable intake or if the family could be physically active together.

### Leading the way: key role of maternity healthcare for migrant women

Women expressed trust for maternity healthcare and its key role in knowledge-building and support for healthy lifestyle behaviors during pregnancy. Women reported feeling comfortable and safe with their pregnancy-monitoring and how maternity healthcare is working in Sweden. However, it was also stated that women that recently arrived in Sweden might experience insecurities regarding contacting and communicating with maternity healthcare due to language barriers or not knowing the structure of maternity healthcare. Women expressed that the healthcare professionals they meet on a regular basis provided support for healthy lifestyle behaviors through recommendations and tips on how women can improve their diet and physical activity. In some cases, recommendations or reinforcement from maternity healthcare could be enough for women to adopt healthier habits.

*[…] I eat and I follow what they have told me. […] Usually I like to eat white bread. But since they have told me what is good, which is now during pregnancy, I try to eat whole meal bread*. **Somali woman, 20 years, interview with interpreter**

In contrast, some women voiced that they lacked information about diet, physical activity and GWG from maternity care, and expressed that receiving information was dependent on whether they asked for the information themselves. Further, it was highlighted that GWG mainly was brought up in maternity healthcare if women already had pre-pregnancy overweight or obesity.

*Yes, they talk about it but not much. Sometimes I feel that you have to take the initiative to talk about it, things like that [diet and physical activity].* **Somali woman, 27 years, interview without interpreter**

Women reported that communication usually works well in maternity healthcare (with or without an interpreter), but it was also highlighted that women might not express if they do not understand information, which could cause worry.

### Speaking my language: app as support for healthy lifestyle behaviors

Women viewed an app in their native language as a promising tool for support throughout pregnancy, and put special emphasis on the credibility of the app since it would be offered through maternity healthcare, thereby increasing the trust for the app. One of the main advantages with an app would be the constant availability of information without the need to translate it.

*It will reduce the hassle of translating if the information was in Swedish and sometimes I would feel tired of the entire process, so then I would be able to read it without thinking about translation. **Arabic woman, 22 years, interview with interpreter***

Women identified similarities between information provided in the app and by maternity healthcare and saw that as something positive as they could use the app to repeat information they already received. An early introduction of the app could be beneficial since information and support for lifestyle behaviors were requested in early pregnancy as the need is large at that time. It could be especially important to receive information and support early on given that the first visit in maternity healthcare usually occurs two months into pregnancy and visits are not frequent in the first trimester.

It was expressed that the app could be a personal guide for women to navigate misinformation from their social environment regarding diet and physical activity, as they would trust the app to have the correct information. Women further requested detailed information on diet (i.e., what foods to eat and avoid and why). They also reported wanting information on how typical traditional dishes could be adapted to be healthier (e.g., to be less heavy in refined carbohydrates and how a balanced meal is composed). Some women also wanted the app to complement maternity healthcare by addressing subjects that could be difficult to talk about, including genital mutilation in relation to delivery and intimacy during pregnancy. In terms of making adaptations to the existing *HealthyMoms* app women suggested that the app should be prolonged to also cover the post-partum period and it was further requested that the app should be more inclusive for the partner.

*I want more, like engagement in the actual app. Like it's not only dedicated for women. It's like a family app, so that both of us can download it and he can have information for him [the partner] there. **Arabic woman, 27 years, interview with interpreter***

Women suggested that the app should include video- and audio material which could reach more women due to difficulties in comprehending written content. Audio content could further be beneficial since women could listen to app content while doing other things at the same time. Some women did not request any cultural adaptations but would be satisfied as long as the app was translated into their native language.

## Discussion

Our findings indicate a considerable need and desire for increased knowledge and information regarding pregnancy and healthy lifestyle behaviors in Arabic- and Somali-speaking women (the two most common migrant groups in Sweden). Although women expressed awareness of the importance of a healthy diet and physical activity, they did not necessarily engage in such behaviors. The results also indicate that maternity healthcare and the social environment have important roles to motivate women to healthier behaviors. Finally, women were positive towards receiving support for healthier behaviors in their native language and viewed a pregnancy app, i.e., *HealthyMoms*, as a useful tool to guide them through pregnancy.

The results in this study complement our previous findings from interviews with healthcare professionals^30^ and together they give a comprehensive view of how lifestyle behaviors can be promoted in Arabic- and Somali-speaking women. We found both similarities and differences when comparing the findings of the studies. For instance, one major similarity was the reported need for increased knowledge regarding lifestyle behaviors (i.e., diet, physical activity and GWG). Further, lifestyle behaviors were expressed by both women and healthcare professionals to be influenced by cultural background and social environment. Similar findings have also been observed in women of child-bearing ages (including pregnancy) in high-income countries^34^ as well as in Somali non-pregnant women in Sweden, where misconceptions about lifestyle behaviors were affected by women's previous life experiences and cultural traditions.^35^ Further, similar to our findings, this study found that women's awareness of health benefits from physical activity did not lead to increased activity levels.^35^ Findings from our study and from healthcare professionals^30^ highlighted the lack of knowledge regarding GWG and possible adverse pregnancy outcomes related to it. In addition, qualitative data in African women living in the UK has similarly shown that cultural beliefs and the social environment can affect how women view GWG.^36^ Moreover, the study reported that conflicting information from the social environment and healthcare could be of concern, which was also expressed by healthcare professionals and women in this study and common misinformation should thereby be addressed in maternity healthcare. Similar findings have previously been demonstrated in a systematic review of migrant women's experiences of maternity healthcare in Europe (qualitative and mixed methods data), where it was reported that healthcare professionals’ advice often are conflicting with advice from the social environment.^37^ Moreover, in our previous interviews with healthcare professionals^30^ it was expressed that women to a large extent trust the social environment over advice from maternity healthcare. This finding is contradictive to what women highlighted in this study, where they expressed a big trust in maternity healthcare and considered it the most reliable source of information. Moreover, healthcare professionals voiced that the *HealthyMoms* app would need cultural adjustments and not only be translated to benefit Arabic and Somali-speaking women^30^ but some women actually expressed that the translation alone would be sufficient, although they provided some suggestions for adaptations of the app (e.g., more audio/video content, and inclusion of the partner). Similar to our findings, a Swedish study in newly arrived migrants (Arabic- and Somali-speaking, non-pregnant population) demonstrated that contradictive information was reported to cause confusion and participants reported difficulties assessing reliable sources.^38^ Moreover, some women in our study expressed challenges navigating webpages with information related to pregnancy and lifestyle behaviors (also including credible webpages recommended by healthcare), which highlight a need for credible sources or mHealth tools that are easily navigated although language skills are lacking. To summarize, maternity healthcare has an important role in guiding women to credible sources, but it is important to be aware of difficulties in navigating webpages for migrant women, especially if language skills are lacking and here a translated app could provide a comprehensive support for healthy lifestyle behaviors in women's native languages.

### Strength and limitations

One of the strengths with this study was the use of purposive sampling to recruit pregnant women through maternity healthcare, which enabled inclusion of women from two of the largest migrant groups in Sweden, i.e., Arabic-speaking countries (i.e., Iraq, Syria) and Somalia.^27^ We were further able to recruit women who had been in Sweden for various time periods (from 6 months up to 18 years) and thereby get a sample of women with diverse knowledge about Swedish society and healthcare experience. Furthermore, our study sample consisted of both nulliparous and multiparous women with a variation in educational levels (from no education to university degree). The variation in sample characteristics provided broad and rich data of the phenomena under investigation.^39^ We did not determine the sample size beforehand, rather, data was collected until the research team perceived that data saturation had been reached, i.e., that the material was rich and broad and considered satisfactory to answer the study objective.^40^ There are also limitations to this study. We conducted interviews over the phone which previously has been described as inferior to in-person interviews.^41^ However, more recent data has indicated that data is similar between in-person and phone interviews^42^ and that telephone interviews can provide rich data.^43^ Moreover, given the COVID-19 pandemic we did not have the option to perform interviews in-person and phone interviews were considered the most viable option. Another potential limitation includes that the researchers analyzing the data were not representative of participants in terms of ethnicity and moreover, findings from the analysis were not checked with the participants, i.e., member checking. However, in this context, it is relevant to note that we are, as a next step of this research, feeding back the information obtained from this qualitative study into the next phase i.e., co-creating the relevant adaptations required together with Arabic- and Somali-speaking women and community workers. Finally, we used an interpreter for most interviews (15 out of 19) and there could therefore be a risk of losing information in the translation. However, insights were similar between interviews (also compared to interviews conducted without an interpreter) which strengthens the credibility of the interpretation. It is also relevant to note that in order to reach non-Swedish speaking women, the use of interpreters was vital in order to capture important views and experiences from non-Swedish speaking women.

We used various strategies to increase the trustworthiness^32,40^ of our study results. Credibility was ensured by recruitment of women from different birth countries, and time spent living in Sweden, and we could thereby get a broadness in experiences. Credibility was further strengthen by investigator triangulation where a rigorous and systematic process was conducted with inspiration from Graneheim and Lundman^32^ when performing the analysis. To specify, data was coded by two researchers (ES and SB) and latent codes were double checked before continuing creating sorting categories and deciding on themes, a process conducted by several members of the research team. In addition, presenting excerpts from interviews further strengthens credibility since it provides insights and increases transparency of results. The use of an interview guide that had been developed by the research team with knowledge in the topics that aimed to be investigated increased dependability. Finally, transferability was enhanced by a careful description of the participating women of our study (including time living in Sweden, birth country, parity, educational level). However, as stated by Graneheim and Lundman,^32^ the transferability of the results to other contexts must be judged by the reader.

### Implications and clinical relevance

Findings from our study highlight the need of maternity healthcare to provide more support for healthy lifestyle behaviors in migrant women. Especially information on dietary habits and GWG seems to be lacking. Moreover, GWG mainly seems to be focused on if women have pre-pregnancy overweight or obesity. Although it is highly important to target women with overweight and obesity, excessive or insufficient GWG regardless of pre-pregnancy BMI are associated with increased risks of adverse pregnancy outcomes for mother and child.^5,6^ Therefore, it is essential for maternity healthcare to inform all women about these risks. Our findings also indicate a clear potential for using mHealth as support for a healthier lifestyle in migrant women during pregnancy and a translated mHealth tool such as the HealthyMoms app has several advantages compared to already existing and available information at e.g., national websites. First, such websites might be difficult to navigate which was also highlighted in our data. Moreover, the HealthyMoms app is grounded in social cognitive theory and have key behavior change techniques including goal setting, registration of key health behaviors (diet and physical activity) as well as GWG and the app additionally provides feedback on registered data. In other words, the HealthyMoms app is more comprehensive than a translated national website for health information. Also, the app has previously been evaluated in a full scale randomized controlled trial, where an intervention effect indeed was observed on diet as well as GWG in women with overweight and obesity who received the app. Clearly, it is of great importance that solutions provided through healthcare are evidence based.

mHealth research and its use in migrant populations are still scarce but more evidence is emerging on how mHealth solutions should be adapted to best reach migrant populations (e.g.,^23,25,26^), also applying for other digital medias.^44^ One of The United Nations Sustainable Development Goals for 2030 is to reduce inequalities within healthcare as well as mortality rates in mothers and infants.^14^ Indeed, mHealth tools supporting a healthy lifestyle during pregnancy that are translated and adapted to a country's most common migrant groups, may play a significant role in achieving these goals. More specifically, our study contributes with important insights on how mHealth solutions could be tailored for migrant women. Firstly, given the importance of the social environment, and that women in our study requested more information for the partner, mHealth solutions for the pregnant population could benefit from being more inclusive in terms of the partner, which is in line with recent findings in other countries^14^ and was also reported in women using the Swedish version of HealthyMoms app.^19^ A potential way of increasing the social support could be by connecting a user profile to the woman's app for the partner to use. Thereby partners could follow the development of the pregnancy, get relevant information related to healthy lifestyle behaviors, get guidance through common misconceptions, and finally get information on how they best could support women to eat healthily and be physically active during pregnancy. Moreover, future research should also focus on exploring how health behaviors could be improved in migrant women by including their partners. Also, a previous review emphasized that migrant father's role during pregnancy need further investigation.^45^ Further, our results showed that women could be motivated by seeing benefits of their health behaviors in their children, indicating that interventions that has a family focus, i.e., family interventions, could be a possible way forward. Secondly, an interesting observation is that some migrant women in our study indicated that they would be satisfied if they got a tool such as the *HealthyMoms* app translated to their native language. Although the women provided some suggestions for additional content, cultural adjustments were not described as pivotal. To the best of our knowledge this is a novel finding, contrasting previous results in healthcare professionals^30^ and we can only speculate why this is. It is possible that women responded in order to meet perceived expectations from researchers (i.e., out of social desirability). Another potential explanation may be that migrant women have low expectations on healthcare and would thus be satisfied with easily accessible information in their native language provided by a credible source. A further explanation could be that pregnancy is similar to all women regardless of cultural background, and therefore an inclusive approach for all pregnant women could be adequate support. Whether we should aim primarily for translations only or more in-depth culturally adapted mHealth tools is a topic for future research and should include different countries and healthcare contexts with migrant populations. Our results also highlight the need of creating mHealth tools in a co-creation process^46^ including both women and healthcare professionals. This work should include both initial perceptions but also ascertain the tools to be sufficiently powerful to create behavior change. Indeed, there is need for more research regarding how cultural adaptations can increase effectiveness of dietary and physical activity interventions.^47^

## Conclusion

Arabic- and Somali-speaking women expressed an increased need of information related to lifestyle behaviors during pregnancy and highlighted the importance of their social environment and maternity healthcare to support such behaviors. A translated pregnancy app was perceived as a helpful tool for healthy lifestyle behaviors and women expressed that translation alone could motivate them to use it. Findings from this study can help guide maternity healthcare on what support to provide migrant women for healthy lifestyle behaviors as well as inform refinement and future development of mHealth tools for pregnant migrant women and thereby mitigate health inequalities in prenatal care. Future research topics include disentangling the degree of cultural adaptations that are required for mHealth lifestyle interventions provided by healthcare.

## Supplemental Material

sj-docx-1-dhj-10.1177_20552076241304045 - Supplemental material for Adapting a pregnancy app (HealthyMoms) to support healthy habits in migrant women—a qualitative study on women's preferences and perceived needs to support health behaviors during pregnancy Supplemental material, sj-docx-1-dhj-10.1177_20552076241304045 for Adapting a pregnancy app (HealthyMoms) to support healthy habits in migrant women—a qualitative study on women's preferences and perceived needs to support health behaviors during pregnancy by Emmie Söderström, Christina Alexandrou, Sara Bressanutti, Johanna Sandborg, Anna-Karin Lindqvist and Marie Löf in DIGITAL HEALTH

sj-docx-2-dhj-10.1177_20552076241304045 - Supplemental material for Adapting a pregnancy app (HealthyMoms) to support healthy habits in migrant women—a qualitative study on women's preferences and perceived needs to support health behaviors during pregnancy Supplemental material, sj-docx-2-dhj-10.1177_20552076241304045 for Adapting a pregnancy app (HealthyMoms) to support healthy habits in migrant women—a qualitative study on women's preferences and perceived needs to support health behaviors during pregnancy by Emmie Söderström, Christina Alexandrou, Sara Bressanutti, Johanna Sandborg, Anna-Karin Lindqvist and Marie Löf in DIGITAL HEALTH
