# Supplementary material for: Adapting a pregnancy app (HealthyMoms) to support healthy habits in migrant women—a qualitative study on women's preferences and perceived needs to support health behaviors during pregnancy
Source: Digit Health. 2024 Dec 12;10:20552076241304045. doi: 10.1177/20552076241304045 (PMC11635882; doi:10.1177/20552076241304045)
Supplement: sj-docx-1-dhj-10.1177_20552076241304045 - Supplemental material for Adapting a pregnancy app (HealthyMoms) to support healthy habits in migrant women—a qualitative study on women's preferences and perceived needs to support health behaviors during pregnancy [file sj-docx-1-dhj-10.1177_20552076241304045.docx]

Supplementary files 1. Interview guide and brief questionnaire.

Söderström et al. Adapting a pregnancy app (HealthyMoms) to support healthy habits in migrant women– A qualitative study on women’s preferences and perceived needs to support health behaviors during pregnancy

# Interview guide

1. If you think about the knowledge you have about pregnancy, where did you get this knowledge from? If you are searching for information about your pregnancy, where would you look for this information? What sources do you think are reliable?
2. What support do you get from your family, friends and your partner during pregnancy? Compared to your life before you got pregnant, has the support changed?
3. Are the recommendations regarding lifestyle behaviors for pregnant women in your home country different from recommendations in Sweden? If yes, in what way?
4. What are your thoughts about weight gain during pregnancy? Is it something you try to affect?
5. What is physical activity to you? What are your view on physical activity and exercise during pregnancy?
6. Have you changed your physical activity level since you became pregnant? If yes, in what way in terms of intensity, duration and frequency?
7. How many meals do you on average eat in a day? How does a typical breakfast/lunch or dinner look for you?
8. How does your eating habits differ from the Swedish food culture? Do you eat the same type of foods, or do you exclude something in your diet that is common to eat in Sweden?
9. What support would you like to have during pregnancy from the healthcare? What would this support look like?
10. If there was a pregnancy app that the maternity health care would offer, is that something you would be interested in using? Why/why not?
11. Would you prefer support during pregnancy through a smartphone app, daily texts (sms) or in some other way?
12. Are you using app or any other support during pregnancy? If yes, what type of tool are you using and what do you like and do not like with it?
13. If you were to decide, what would be important for you to include in a pregnancy app?

Questions related to the HealthyMoms app (asked after a thorough description of the app content)

1. What are your thoughts about the content in the app? Do you have suggestions on content that should be added/removed?
2. Would you prefer the app to have information in text, pictures, videos or audio?
3. In the existing version of the app (HealthyMoms) there is a pregnancy calendar in which you can read about what is happenings with the mother, the growing baby and there is also information for the other parent. Is this something you are interested in to have in a pregnancy app? Why/why not?
4. If you had the HealthyMoms app in Swedish and it was translated to your native language, would you use the app more or less and why? What cultural adjustments would be necessary to make the app useful to you?
5. If you think about yourself and other pregnant women in your surroundings, would there be an interest in using an app that is similar to the HealthyMoms app. Why/why not?

# Backgroud questions

- 1. Number of children: Children ages:
  2. Year of birth:
  3. Birth country:
  4. Mother’s birth country:
  5. Father’s birth country:
  6. Education:
     1. Elementary school
     2. High school (2 years), vocational training or equivalent
     3. High school (at least 3 years)
     4. University degree
     5. Other (e.g., no school):
